# Retrospective evaluation of pregnant women with celiac disease

**DOI:** 10.4274/jtgga.2016.0198

**Published:** 2017-03-01

**Authors:** Kemal Beksaç, Gökçen Örgül, Murat Çağan, Ergun Karaağaoğlu, Serap Arslan, Mehmet Sinan Beksaç

**Affiliations:** 1 Department of General Surgery, Ankara Oncology Hospital, Ankara, Turkey; 2 Division of Perinatology of the Department of Obstetrics and Gynecology, Ankara Oncology Hospital, Ankara, Turkey; 3 Department of Biostatistics, Ankara Oncology Hospital, Ankara, Turkey; 4 Department of Gastroenterology, Hacettepe University Faculty of Medicine, Ankara, Turkey

**Keywords:** celiac disease, gluten-free diet, Pregnancy, perinatal morbidity, low-molecular- weight heparin, low-dose corticosteroids

## Abstract

**Objective:**

To show celiac disease (CD) and its poor pregnancy outcome relationship, and to demonstrate the importance of a gluten-free diet together with low-dose low-molecular-weight heparin (LMWH) and low-dose corticosteroid (LDC) in the management of pregnancies with CD.

**Material and Methods:**

This study consisted of 2 groups of patients. Six patients with CD (control group) on a gluten-free diet were monitored during their first pregnancies within the framework of antenatal care program and their pregnancy outcomes were compared with eight poorly-treated pregnant patients with CD (study group) who were referred from other medical institutions. LMWH (enoxaparine 1x2000 Anti-XA IU/0.2 mL/day), and LDC (methylprednisolone 1x4 mg p.o/day) were used in the control group. Their obstetric histories and outcomes of their last pregnancies were compared. The patients’ obstetric risk levels were evaluated using the “Beksac Obstetrics Index” (BOI).

**Results:**

There were miscarriages in 50% of the study group. There were also 50% and 75% preterm deliveries in the control and study groups, respectively. The BOI of the study group was significantly worse than the control group (1.31 vs. 0.31±0.21, p<0.01). There were no statistically significant differences between age (24±4.7 vs 31.7±6 years, p=0.448), gestational day of birth (259.3±8.5 vs 246.6±24.3), birthweight (2691±698 vs 2262±359 g, p=0.394), and cesarean section rates (p=0.118).

**Conclusion:**

CD is a risk factor for adverse pregnancy outcome. Miscarriage and preterm labor are critical complications in pregnancies complicated by CD. A gluten-free diet is important in the treatment. LMWH and LDC seem to be helpful in the management of pregnant women with CD.

## INTRODUCTION

Celiac disease (CD) is a small intestinal enteropathy, which is activated by gluten ingestion in patients with a genetic background (1). The prevalence of the disease is around 1% (2, 3). “Gluten, gluten-specific T-cells, the major histocompatibility complex antigen HLA-DQ, and transglutaminase type 2 (TG2)” are the main actors of this disorder. It has been reported that repeated miscarriages and obstetric complications are more frequent in patients with CD (4, 5).

CD is an autoimmune disorder characterized by circulating anti-TG2 autoantibodies (6, 7). It has been reported that anti-TG2 antibodies act negatively on endometrial receptivity and impair decidual angiogenesis together with interstitial trophoblast migration along with various mechanisms (8). It has also been reported that the chorionic villus (materno-fetal interface) is one of the main targets of anti-TG2 autoantibodies and these antibodies directly attack syncytiotrophoblasts (9). In other words, impaired endometrial receptivity and disturbed syncytiotrophoblastic apoptosis might be the main causes of impaired fetal perfusion (intrauterine hypoxia) and poor obstetric outcome.

In this report, we compared the pregnancy outcomes of 6 primigravida patients at complete remission on a gluten-free diet and 8 referred patients with CD who had various gestational symptoms in terms of obstetric outcome.

## MATERIAL AND METHODS

This retrospective study was conducted at the Department of Obstetrics and Gynecology, Hacettepe University, between March 2014 and March 2016. There were two groups of patients. The control group comprised six patients with CD on gluten-free diet who were monitored during their first pregnancies. Their pregnancy outcomes were compared with eight pregnant patients (study group) with CD from other medical institutions who had been referred because they had not adhered to their recommended gluten-free diet. All patients were diagnosed with CD before their pregnancies.

We used the Beksac Obstetrics Index (BOI), which is an obstetrics index for the assessment of risk levels of high-risk pregnancy groups [(number of alive children + π/10)/Gravida], in order to compare these two groups (π=3.14) (10).

Patients in the control group were followed up under the “autoimmune disorders in pregnancy” protocol within the special antenatal care program of the Division of Perinatal Medicine. Laboratory tests were performed (complete blood count, liver function enzymes, antithrombin-III and activated protein-C activities, complement 3 and 4, blood glucose level, hereditary thrombophilia-related polymorphisms, antibodies such as antinuclear antibodies, antiphospholipid antibodies, anti-smooth muscle antibodies, anti-double stranded DNA, and others according to individual differences), and necessary precautions were undertaken. Low-dose low-molecular-weight heparin (LMWH) (enoxaparine 1x2000 Anti-XA IU/0.2 mL/day), and low-dose-corticosteroid (methylprednisolone 1x4 mg p.o/day) were used together with CD-specific treatment in the control group. In one patient, low-dose corticosteroid (LDC) was used alone without LMWH. Study group patients who did not have an abortion received standard CD treatment at the outpatient clinic.

The Statistical Package for the Social Sciences version 17 (IBM SPSS Statistics, Chicago, IL, USA) was used for data analysis. Pearson’s Chi-square and Fisher’s exact test were used for categorical variables and the t-test was used for continuous variables.

This study was performed in compliance with the ethics principles of the university board and those of the national committee. All patients were informed about the study and signed informed consent. The non-interventional clinical research ethics board approval number is GO 16/100 (2016).

## RESULTS

The demographics of the patients are given in Table 1 and 2. The mean age of the control group patients was 24±4.7 years and the mean age of patients in the study group was 31.7±6 years (p=0.448). The control group consisted solely of primigravid patients. They were known to have CD before their pregnancies, referred to obstetricians early in their pregnancies, and precautions were taken early to ensure a successful outcome. All patients referred from other clinics were multigravida patients and almost all of them had previous abortions. Half of this group’s pregnancies ended with abortion.

Half of the pregnancies referred from other medical institutions ended with abortion. The mean gestational day of birth was 259.3±8.5 in the study group and 246.6±24.3 in the control group. There were no statistically significant differences (p=0.697).

Birthweights were similar in both groups. Mean birthweight in control group was 2691±698 and 2262±359 in study group (p=0.394).

Four of six patients in the control group and three of four patients in non-treatment group gave birth via cesarean section. There were no statistically significant difference regarding their delivery routes (p=0.118).

The patients’ obstetrics risk levels were evaluated using the BOI. The BOIs of the entire control group was 1.31 because they were all primigravida patients. The mean BOI value of the non-treatment group patients was 0.31±0.21. This difference was statistically significant (p=0.001).

## DISCUSSION

CD is an autoimmune small intestinal enteropathy, which is activated by dietary gluten (cereal prolamins) and its incidence is about 1% (1, 3). Increased risk of pregnancy failure and obstetric complications has been reported in patients with CD (1, 4, 5). It has been reported that up to 50% of women with untreated CD have a history of miscarriage and other unfavorable pregnancy outcomes, which is similar to our findings (11). Untreated patients with CD also have a higher risk of developing intrauterine growth retardation, low birthweight, stillbirth, pre-term birth, and small-for-gestational-age babies compared with pregnancies with treated CD (11, 12). In our study, there were 50% and 75% preterm deliveries in the control and study groups, respectively.

Anti-TG2 autoantibodies were reported to be the main source of placenta-specific inflammatory process in patients with CD, which resulted in intrauterine hypoxia and impaired fetal perfusion (6, 7). Impaired apoptosis of syncytiotrophoblasts and disturbed endometrial receptivity by circulating anti-TG2 antibodies seems to be the reason for these implantation and placentation disorders (1, 8, 9).

In our small series, we demonstrated that patients with active CD who were not on a proper gluten-free diet experienced poor pregnancy outcomes, and their BOIs were statistically significantly lower than patients with CD on a gluten-free diet. It has been reported that anti-TG2 plasma levels decreased when CD went into complete remission with a gluten-free diet (13). Control of circulating anti-TG2 antibodies should be the goal during perinatal surveillance. This may be a rationale in for prophylactic use of LDCs in certain cases, especially when the complement system is activated.

On the other hand, the rising prevalence of venous thromboembolism among patients with inflammatory bowel disease and autoimmune diseases should be our concern during the management of these pathologies (14). The importance of prophylactic low-dose low- molecular-weight heparin use in diseases such as CD in necessary cases lies in the following: anti-TG2 antibodies bind to human endometrial endothelial cells and impair endometrial angiogenesis by inhibiting the activation of matrix metalloprotease-2 (MMP-2) activity (1, 8). Thus, these biologic changes may be responsible for the induction of venous thromboembolic events. The other possible mechanism for the endothelial injury of vascular structures around the materno-fetal interface (chorionic villae) is the direct attack of anti-TG2 antibodies on endothelial cells of spiral veins, together with endovascular trophoblasts covering and occluding the tip of spiral arteries, which are the opening to the intervillous space, and the syncytiotrophoblasts that cover the outer surface of chorionic villae. Autoimmune antibody positivity should be taken into consideration as a risk factor for poor pregnancy outcome (15).

The final goal should be the elimination of anti-TG2 antibodies through dietary precautions and/or suppression of antibodies by the preventive use of LDCs in necessary cases, especially when the complement system is activated. Endothelial injury of vascular structures should be eliminated. Low-dose LMWH might be critical in cases with antithrombin III and activated protein-C activity changes. Elimination of thrombus formation is also critical to prevent secondary activation of the complement system, which itself may also give harm to surrounding tissues. In our series, we used low-dose LMWH in 5 of the 6 CD patients in the control group (patients with CD under long-term follow-up) due to active protein-C and antithrombin III activity changes. All of these patients delivered successfully without perinatal mortality and severe morbidity. Three of these patients had preterm deliveries without important neonatal complications.

We must also remember that the destruction of chorionic villae by these toxic materials (anti-TG2 antibodies, cell degregades of endothelial cells of spiral veins and complement system proteins) will result in the release of fetal cell degregades (syncytiotrophoblasts) into the maternal circulation and cause a graft-versus-host-like inflammatory process in the placenta. All these patho-biologic events are most probably the reason of impaired fetal perfusion and hypoxia, and these might be the reason of increased obstetric complications such as miscarriage, intrauterine growth retardations, preterm deliveries, and possibly preeclampsia in patients with CD, as observed in the uncontrolled patients with CD of our clinical series. We believe that patient-specific individualized management is essential in pregnancies with CD and dietary control is necessary to provide better pregnancy outcomes. LMWH and LDC seem to be helpful in the management of pregnancies with CD.

Further studies are necessary in this field to understand CD in pregnancy. This study is limited by the low number of patients.

## Figures and Tables

**Table 1:**
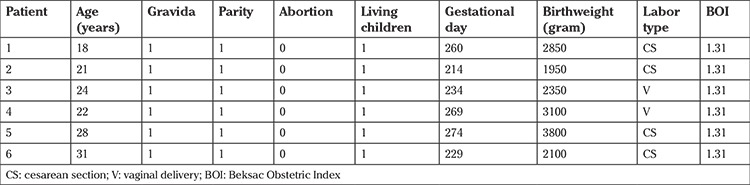
Demographic findings of patients in their last pregnancies (under medical treatment)

**Table 2:**
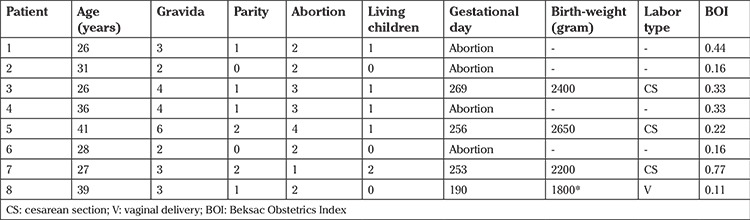
Demographic findings of patients in their last pregnancies (without any treatment)
